# Patient blood management programs: how to spread the word?

**DOI:** 10.1186/s13584-018-0204-5

**Published:** 2018-01-15

**Authors:** Shoshana Revel-Vilk, Mira Naamad

**Affiliations:** 1 Pediatric Hematology, Shaare Zedek Medical Center, affiliated with Hebrew University Medical School, Jerusalem, Israel; 2 Gaucher Unit, Shaare Zedek Medical Center, Jerusalem, Israel; 3 Blood Bank, Shaare Zedek Medical Center, Jerusalem, Israel

## Abstract

Red blood cell (RBC) transfusions save lives and improve health; however, unnecessary transfusion practice exposes patients to immediate and long-term negative consequences. Indirect consequences of unnecessary transfusions are the reduced availability of RBC units for patients who are in need. Accumulating evidence shows that *restricting* RBC transfusions improves outcomes and current guidelines suggest limiting RBC transfusion to the minimum number of units required to relieve symptoms of anemia or to return the patient to a safe hemoglobin range (7–8 g/dl in stable, non-cardiac inpatients). Still, studies show that there is over-utilization of RBC transfusion, partly due to low level of knowledge of physicians regarding restrictive RBC transfusion policy across a broad range of professions and specialties. Patient blood management (PBM) programs have been developed to promote clear hospital transfusion guidelines, strive for optimization of patient hemoglobin and iron stores and, most importantly, improve education regarding restrictive RBC policy. Understanding what and where the gaps of knowledge are, as was done in the study by Dr. Koren and his colleagues, is an important step for developing effective PBM programs.

## Background

Restrictive blood management policy is a relatively new concept. For many decades, red blood cell (RBC) transfusion was used liberally, without specific threshold triggers and with no evidence based data of benefits or risks. With the accumulating evidence that *restricting* RBC transfusions improves patients’ outcomes the policy has gradually changed. Pooled results from 3 trials with 2364 participants showed that a restrictive hemoglobin (Hb) transfusion trigger of Hb < 7 g/dl resulted in reduced mortality and hospital-related morbidity compared with a more liberal strategy; the number needed to treat (NNT) with a restrictive strategy (Hb < 7 g/dl) to prevent 1 death was 33 [1]. Transfusion strategies showing the benefit of *restricting* RBC transfusions have been evaluated in various settings including adult critical care [2, 3], pediatric critical care [4] and in patients with acute upper gastrointestinal bleeding [5]. However, it is important to emphasize that given that the risks and benefits from blood transfusion are not straightforward, it is plausible that optimal transfusion thresholds may vary based upon the level of risk and underlying medical disorder.

Guidelines for RBC transfusion in stable, non-bleeding patients were developed and published based on a synthesis of existing clinical evidence, practice guidelines, and institutional preferences [6]. Stable, non-bleeding medical and surgical inpatients are considered candidates for RBC transfusion when the Hb level is ≤7 g/dl. Transfusion should be considered for inpatients with active, acute coronary syndromes with a Hb level ≤ 8 g/dl, with exceptions including low oxygen saturation, end-organ ischemia, ongoing bleeding, and hypotension [7]. Critical care medical and surgical adult inpatients being treated for sepsis during the first 6 h of resuscitation may be transfused with a Hb level ≤ 10 g/dl. All RBC transfusions in non-bleeding inpatients should be ordered as single units. If transfusion is indicated based on Hb level, post-transfusion Hb must be obtained before ordering additional units [6]. One of the five first recommendations of The American Society of Hematology (ASH) Choosing Wisely® committee focused on avoiding liberal RBC transfusion [8]. The specific recommendation was that in situations where transfusion of RBCs is necessary, transfusion should be limited to the minimum number of units required to relieve symptoms of anemia or to return the patient to a safe hemoglobin range (7–8 g/dl in stable, non-cardiac inpatients).

## Patient blood management

Patient blood management (PBM) programs have been developed worldwide in order to optimize the utilization of blood components and, as a result, an up to 40% reduction in RBC units transfused per patient has been achieved [9–17]. Lack of such hospital PBM programs results in an extensive liberal RBC transfusion practice, as was shown in a large Danish study [18] and in a study we performed in three hospitals in Jerusalem, Israel [19]. In order to succeed with reducing the utilization of RBC units, the PBM program needs to include several important elements:

1. Clear hospital transfusion guidelines including single unit transfusion policy, laboratory “gatekeeping” and the use of an electronic ordering system for blood products (identifying the clinician who ordered blood products is important for feedback and audit).
2. Optimization of patient hemoglobin and iron stores by appropriate diagnosis and treatment of anemia (especially prior to surgeries/procedures), optimization of hemostasis, and minimization of iatrogenic blood loss (i.e. reduction of patient blood sampling, reduction of surgery-related blood loss)
3. Implementing a comprehensive information and consent form outlining the risks and benefits of RBC transfusion, and requiring signatures from both patient and clinician, thus enhancing general awareness among clinicians regarding the adverse events associated with allogeneic blood transfusions.
4. Education.

## Clinicians’ knowledge of patient blood management

In their interesting paper which was recently published in IJHPR, Dr. Koren and his colleagues address the issue of clinicians’ knowledge regarding RBC use, specifically the knowledge concerning restrictive blood management policy [20]. As correctly stated by the authors, the lack of knowledge in the field of transfusion medicine may play an important role in over-utilization of RBC transfusion. Understanding what and where the gaps of knowledge are is an important step for developing an effective educational program for PBM. In their cohort of 79 physicians working in the surgical and internal medicine department at the Galilee Medical Center in Israel, the overall transfusion- related knowledge was found to be average (mean score was 47.8 ± 18.6) and to differ between fields of specialty, i.e. internal medicine physicians showing a greater level of knowledge compared to surgeons, and by level of seniority. No differences in response score was found regarding indications for transfusion. Knowledge regarding familiarity with restrictive blood management was similarly low and again differed between fields of specialty, i.e. internal medicine physicians demonstrating a higher level compared to surgeons, and senior physicians a higher level than juniors. Interestingly, in a study we performed in three hospitals in the Jerusalem area, the results were similar; Familiarity with the term “restrictive transfusion” was greater among senior physicians compared to interns/residents [OR 3.95 (95% CI 2.09–7.47)] and among internists compared to surgeons [OR 2.35 (95% CI 1.26–4.37)]. Inadequate knowledge regarding the principals of PBM was also reported among clinicians working in seven European hospitals [21], 1242 physicians from Iran [22], and 474 residents from 23 programs in the USA [23]. Importantly, the majority of residents (65%) stated that additional transfusion medicine training may be “very” or “extremely” helpful [23]. Although RBC transfusion is one of the most common procedures performed in hospitals [24], it is surprising and disappointing that the training of medical students and residents in the field of transfusion medicine is lacking [25, 26].

## So what should and can be done now?

The first step is to achieve nationwide agreement to adopt a *restrictive* RBC transfusion policy. This was recently done when the Hematology Choosing Wisely® committee of the Israel Medical Association adopted a statement similar to the ASH document, encouraging the use of no more than the minimum number of RBC units necessary to relieve symptoms of anemia or to return a patient to a safe hemoglobin range (7 to 8 g/dL in stable, in-patients) (www.ima.org.il).

The next step is to establish PBM programs. The Ministry of Health has issued a call to open a Blood Bank Committee in all Israeli Hospitals with the aim to write and follow in-house RBC transfusions guidelines. These committees could be the base for in-hospital PBM program using a recently published comprehensive working template encompassing over 100 different measures [27]. Implementation of these programs requires a team approach that can be promoted by hematologists and transfusion medicine specialists; however, the involvement of specific specialties, mainly anesthetics, surgery, and intensive care, is essential. The programs should be proactive, patient-centered, and led by hospital key leaders, who should play a central role in domains of communication, education, and documentation.

Extensive educational programs of lectures, workshops, E-learning course, etc. at the undergraduate and postgraduate levels of both medical and nursing staff is the main component of success; and as was shown by Koren, et al. and others, a component that is currently significantly lacking. This educational effort may be undertaken locally (i.e.in and by the hospital or university), but would probably achieve better results if done on a national basis. Introduction of a validated exam can be used to determine knowledge deficits and assist in the design of curricula to improve blood product utilization [28].

In conclusion, the knowledge of physicians (and nurses) regarding restrictive RBC transfusion policy is still a major issue across a broad range of professions and specialties. The need to establish a PBM in hospitals and the need for improved education is clear. Since restrictive RBC transfusion improves survival and reduces cost, the investment needed in education of medical personal will likely also be prove to be cost-effective.

## Abbreviations

NNT: Number needed to treat
PBM: Patient blood management
RBC: Red blood cell
